# Errors in Aerosol Inhaler Use and Their Effects on Maternal and Fetal Outcomes among Pregnant Asthmatic Women (Subanalysis from QAKCOP Study)

**DOI:** 10.1155/2018/7649629

**Published:** 2018-12-18

**Authors:** Wanis H. Ibrahim, Fatima Rasul, Mushtaq Ahmad, Abeer S. Bajwa, Laith I. Alamlih, Anam M. El Arabi, Dhabia Al-Mohannadi, Mohammed Y. Siddiqui, Israa S. Al-Sheikh, Azdin A. Ibrahim

**Affiliations:** ^1^Senior Consultant Pulmonologist and Professor of Clinical Medicine, Department of Medicine, Hamad General Hospital and Weill-Cornell Medical College, Doha, Qatar; ^2^Consultant Physician, Department of Medicine, Hamad General Hospital, Doha, Qatar; ^3^Pulmonary Fellow, Department of Medicine, Hamad General Hospital, Doha, Qatar; ^4^Medical Resident, Department of Medicine, Hamad General Hospital, Doha, Qatar; ^5^Medical Fellow, Department of Medicine, Hamad General Hospital, Doha, Qatar; ^6^Sr. Consultant Physician, Department of Medicine, Hamad General Hospital, Doha, Qatar; ^7^Internal Medicine Resident, Department of Medicine, Hamad General Hospital, Doha, Qatar; ^8^Internal Medicine Resident, Hamad General Hospital, Doha, Qatar; ^9^Gynecologist and Obstetrician, St. Josefs Hospital, Wiesbaden, Germany

## Abstract

Data on inhaler technique and its effects on maternal and fetal outcomes during pregnancy are seldom reported. The primary objective of this study was to evaluate inhaler technique and identify errors in inhaler use among pregnant women with asthma. Secondary objectives were to identify factors associated with poor inhaler technique and study the association between inhaler technique and maternal and fetal outcomes. This was a cross-sectional, face-to-face, prospective study of 80 pregnant women with physician-diagnosed asthma. Seventy-three and 41 asthmatic pregnant women reported using pressurized metered dose inhalers (pMDIs) and dry powder inhalers (DPIs), respectively. Overall, wrong inhaler technique was observed in 47 (64.4%) subjects. Among pMDI users, correct inhaler use was observed in only 26/73 (35.6%) of the patients, with lack of coordination between inhalation and generation of the aerosol and failure to breathe out gently before using the inhaler, being the most common errors. Among DPI users, 21 (51.2%) demonstrated correct inhaler use, with failure to perform a breath-hold for 10 seconds after inhaling the powder and to exhale gently before using the inhaler being the most common errors. Significant associations between inhaler technique and patient's understanding of asthma medications and the kind of follow-up clinic (respiratory versus nonrespiratory clinic) were found. No significant associations between inhaler technique and various maternal and fetal outcomes or asthma control were found. In conclusion, improper inhalation technique is significantly prevalent in pregnant asthmatic women, particularly among those being followed in nonspecialized respiratory clinics. The lack of significant association between the inhaler technique and asthma control (and hence maternal and fetal outcomes) may simply reflect the high prevalence of uncontrolled asthma and significant contribution of other barriers to poor asthma control in the current patient's cohort. Multidisciplinary management of asthma during pregnancy with particular emphasis on patient's education is imperative.

## 1. Background

Asthma is a serious global health problem that imposes a great burden on healthcare systems and patients. It affects 1–18% of the population in different countries, and its prevalence is increasing in many other countries [1]. The prevalence of asthma during pregnancy may be higher than previously estimated and appears to be continuing to increase [2, 3]. Inadequate asthma control during pregnancy is associated with a significant risk for several adverse pregnancy outcomes including perinatal death, intrauterine growth retardation, preeclampsia, premature delivery, and low birth weight [4–7]. Optimal management of asthma during pregnancy is crucial for protecting both mother and fetus. Inhaled medications are the cornerstone treatment of patients with asthma. The use of inhaled therapy has a number of advantages over systemic (oral or intravenous) administration. It allows a smaller dose to be administered, a faster onset of action, and fewer systemic side effects [8–10]. There is a wide variety of different inhalers currently in the market; they can be broadly classified into pressurized metered dose (pMDI), dry powder (DPI), breath-actuated metered dose (BA-MDI), and soft mist inhalers [8]. In day-to-day asthma care practice, the most commonly used devices are the pMDIs and the DPIs. pMDIs are portable, convenient, and multidose devices. These advantages have made them very popular and most widely used devices for inhalation therapy in asthma [11]. Poor inhaler technique has been shown in multiple studies to be significantly associated with poor asthma control, increased risk of exacerbation, and hospitalization even after statistical adjustment for age, smoking, inhaler device type, and self-reported adherence [12, 13]. Furthermore, recent studies have unveiled the health-economic burden of poor inhaler technique in the developed world. Lewis et al. estimated a cost of €782 million attributable to poor inhalation technique across three countries (the UK, Spain, and Sweden) for the two most commonly used DPIs in 2015 [14]. Hence, assessing and emphasizing proper inhaler technique during each asthma-related health visit is a cornerstone step of asthma management and has been emphasized in all asthma guidelines [1, 15]. The correct use of pMDIs entitles performing all the following 5 steps [16]: (1) removing cap and shaking the inhaler, (2) breathing out gently before using the inhaler, (3) putting the mouthpiece in the mouth and applying good seal with the lips, (4) pressing the canister down at start of inspiration and continuing to inhale slowly and deeply, and (5) performing a breath-hold for at least 10 seconds. For DPIs, performance of all the following steps defines a correct use [16]: (1) breathing out gently before using the inhaler, (2) placing the inhaler in the mouth and creating an adequate seal with the lips, (3) deep and forceful inhalation of the powder, and (4) removing the inhaler from the mouth and performing a breath-hold for 10 seconds. Incorrect use is defined as the failure to perform at least one of these steps [16]. Different patient's characteristics can influence the correct use of inhalers including age, educational attainment, race, and patient's understanding of asthma and its treatment [17–19]. Historically, some errors were identified as “critical” or “essential” based on an assumption that if they are not performed correctly, little or no medication reaches the lungs [20]. Among the most important critical errors for pMDI use are removing mouthpiece cover and shaking the device and coordination between device actuation and inhalation, and for DPI use, the most important critical errors are removing/opening mouthpiece and inhaling forcefully and deeply [20]. Recently, the CRITIKAL study provided the first evidence-based identification of the effects of such critical errors on asthma control and exacerbations [21]. In the CRITIKAL study, failure of performing deep and forceful inhalation among DPI users and lack of coordination between device actuation and inhalation among pMDI users led to a greater likelihood of uncontrolled symptoms and a higher rate of exacerbations [21]. We have shown in the QAKCOP study that tremendous improvements in maternal and fetal health care and socioeconomic status did not reflect on asthma control and care during pregnancy in this wealthy nation [22]. About 65% of asthmatic pregnant women in the QAKCOP study had uncontrolled asthma during their pregnancy, and the inhaler technique was incorrect in 64.4% [22]. Nevertheless, data on inhaler technique and the effects of wrong technique on maternal and fetal outcomes during pregnancy are seldom reported in the medical literature. The primary objective of this study was to evaluate the inhaler technique and identify errors in inhaler use among pregnant women with asthma. Secondary objectives were to identify factors associated with the poor inhaler technique and study the association between the inhaler technique and maternal and fetal outcomes (including preterm labor, miscarriage/abortion, preeclampsia/eclampsia, antepartum/postpartum hemorrhage, rate of cesarean section, development of maternal asthma symptoms during delivery, intrauterine growth retardation, congenital anomalies, low birth weight, and neonatal respiratory distress).

## 2. Methods

This was a cross-sectional, face-to-face, prospective study of 80 pregnant women with physician-diagnosed asthma. The study was approved by the Institutional Review Board of Hamad Medical Corporation (Doha, Qatar) (IRB No. 13245/13). The study was conducted from January 2014 to December 2016. Inclusion criteria included randomly selected (systematic random selection) pregnant women with physician-diagnosed asthma who were using inhaler therapy and attended women's hospital and Hamad General Hospital (the largest tertiary hospital) in Qatar during the study period. The study settings included Hamad General Hospital outpatient medical and respiratory clinics and outpatient and inpatient settings of women's hospital. All study subjects were presented verbally and in writing with detailed information about the study and its objectives.

### 2.1. Assessment of Inhaler Technique

Participants who provided informed consent were interviewed face-to-face by one of the authors of this report for an average of 30 minutes and were requested to demonstrate their inhaler technique to the interviewer using their own inhalers. The Global Initiative for Asthma (GINA) criteria mentioned in the introduction section of this paper were used to determine the correct use of inhaler [16]. None of the patients who were invited to participate declined, yielding a 100% participation rate.

### 2.2. Assessment of Asthma Control

We used the Asthma Control Test (ACT) to assess the level of asthma control in the study subjects. The ACT has been previously validated during pregnancy and demonstrated good internal consistency and was responsive to changes in asthma course. Significant associations between asthma control during pregnancy by the Global Initiative for Asthma (GINA) classification and ACT have also been demonstrated [23–25]. The ACT scores range from 5 (poor control of asthma) to 25 (complete control of asthma). An ACT score >19 indicates controlled asthma [25].

### 2.3. Follow-Up of Participants

Following the initial interview mentioned above, participants were interviewed again via phone at 6 months postpartum to assess details of asthma course and maternal and fetal outcomes of pregnancy. To confirm the accuracy of information provided by participants at this time and minimize recall bias, electronic health records were also reviewed to identify maternal asthma symptoms during labor, maternal and fetal complications at birth, weight of the baby at birth, and the presence of congenital malformations.

### 2.4. Statistical Analysis

Qualitative and quantitative data were expressed as the frequency with percentage and mean SD with median and range. Descriptive statistics were used to summarize demographic and all other clinical characteristics of the participants. Associations between at least 2 qualitative or categorical variables were assessed using *χ*^2^ test. For small cell frequencies, *χ*^2^ test with a continuity correction factor or the Fisher exact test was applied. Pictorial presentations of the key results were made using appropriate statistical graphs. A two-sided *P* value less than 0.05 was considered statistically significant. All statistical analyses were performed using SPSS 22.0 (SPSS, Inc., Chicago, Illinois).

### 2.5. Quality Assurance

Prior to starting the study, all physicians involved in conducting interviews with patients received training sessions on how to conduct the interview, asthma guidelines (GINA), and the correct use of inhalers.

## 3. Results

The characteristics of the study population are summarized in Table 1. Seventy-three and 41 asthmatic pregnant women reported using pMDIs and DPIs, respectively, in the current study. Salbutamol inhaler (when needed) was used by the 73 PMDI users. DPIs used by patients included salmeterol/fluticasone propionate (Seretide Diskus®, GlaxoSmithKline, United Kingdom) (26/41), budesonide/formoterol (Symbicort® Turbuhaler, AstraZeneca, United Kingdom) (13/41), and Budesonide DPI (Pulmicort® Turbuhaler, AstraZeneca, United Kingdom) (2/41). Overall, wrong inhaler technique was observed in 47 (64.4%) subjects. Among patients who were using pMDIs, correct inhaler use was observed in only 26/73 (35.6%). The most commonly observed error with the use of pMDIs was lack of coordination between inhalation and generation of the aerosol (observed with 68.1% of patients), followed by failure to breathe out gently before using the inhaler (61.7%) (Table 2). Among patients who were using DPIs, 21 (51.2%) demonstrated correct inhaler use. The most commonly encountered error with the use of DPIs was the failure to perform a breath-hold for 10 seconds after inhaling the powder (65%) followed by failure to exhale gently before using the inhaler (55%) (Table 2). We found significant associations between poor inhaler technique and patient's understanding of types of asthma medications (*P*=0.002) and the kind of clinic in which asthma follow-up was conducted (respiratory versus nonrespiratory clinic) (*P*=0.030) (Table 3). We did not find significant associations between the inhaler technique and various maternal and fetal outcomes (Table 4). Interestingly, unlike asthma in general population in this country, we did not find a significant association between the poor inhaler technique and level of asthma control in pregnant women, which may reflect the multiple factors contributing to poor asthma control during pregnancy [22, 26]. None of the patients was using an aerochamber/spacer.

## 4. Discussion

There are different factors that influence aerosol deposition into the lungs. The technique of inhaler use by the patient is an important patient-related factor. Even with a perfect inhalation technique, almost one-third of the delivered inhaled dose has impacts on the oropharynx when using inhaler devices [27, 28]. With poor inhalation technique, this wasted amount could be further magnified. Hence, following correct inhaler technique steps is crucial to minimize further oropharyngeal deposition and ensure adequate dose delivery to the lungs. When using pMDIs, slow inhalation (over 4-5 seconds in an adult) after a deep exhalation helps to minimize such deposition in the upper airway and enhance delivery of the drug to the lungs. On the contrary, when using DPIs, the patient has to inhale as deeply and hard as possible to overcome the internal resistance to flow and generate the aerosol for inhalation. DPIs also require turbulent energy to deaggregate their formulations and produce a fine-particle dose during the inhalation maneuver. The greater the energy imparted by the patient's inspiratory flow rate, the more effective the particle deaggregation [29, 30]. Coordination between inhalation and generation of the aerosol, so that inhaler should be activated just after onset of inspiration, is crucial to increase drug deposition in the lung during pMDI use [27, 28, 31–33]. Breath-holding further facilitates sedimentation and residence time and thus enhances diffusion by Brownian motion and deposition in the peripheral airways [28, 31]. Using radiolabelled Teflon particles with a mass median aerodynamic diameter of 3.2 microns, Newman et al. found that a 10-second compared with a 4-second breath-hold significantly increased lung deposition of the particles [33]. In many pMDIs (particularly old chlorofluorocarbon (CFC)), the active drug is present as a micronized suspension rather than a solution. As a result, failure to shake the device either before use or between successive dosages can result in improper dispersion or “settling” of the suspension in the propellant, which was clearly shown in studies to reduce the delivery of both *β*2-agonists and corticosteroids by up to 50% [34, 35]. Although modern pMDIs contain hydrofluoroalkane (HFA) (solution) instead of CFC, nevertheless, not all of these devices have the active drugs in true solution. Therefore, shaking of the device continues to be an important step when using pMDIs [34–37]. The finding of a high rate of incorrect inhaler use among patients in the current study concurs with the findings from previous studies that revealed a global suboptimal inhaler technique among asthmatic patients. In a study from the UK, Hardwell et al. found that over 85% of asthma patients were unable to use their pMDI devices correctly at the start of the study. Furthermore, despite training, majority of symptomatic asthma patients in their study population continued to be unable to use their prescribed pMDIs correctly [38]. In a study from a neighbor country, 73% of pMDI users and 92% of DPI users committed at least one critical error [39]. A recent systematic review identified an overall prevalence of correct inhaler use in only 31% of patients using inhalers [40]. Our study also identified poor coordination between inhalation and generation of the aerosol and failure to exhale before firing as the most commonly encountered errors with pMDI use. Multiple previous studies also identified poor coordination of activation of the pMDI and inspiration to be the most common critical error of pMDI use. In a study of over a thousand out-patients attending hospital during a three-month period, Crompton observed 51% of those patients to have difficulty in coordinating aerosol release with inspiration [41]. Melani et al. reported that 50% of pMDI users in their study failed to exhale before actuation of the device [13]. A recent systematic review that evaluated errors in inhaler use revealed that the most frequent pMDI errors were in coordination (45%), speed and depth of inspiration (44%), and no postinhalation breath-hold (46%). The most common errors for DPI use were lack of full expiration before inhalation (46%) and lack of postinhalation breath-hold (37%) [40]. Contrary to public belief, patients do not always find DPIs easier to use or use them more efficiently. In a study of 1644 adult patients, critical errors were observed in 12% of patients using pMDIs, 35% of patients using DPI/Accuhaler, and 44% using DPI/Turbohaler [13, 28]. While the main disadvantage of pMDIs is the need of coordination between pressing down the canister and inhaling the medication, DPIs have the disadvantage of requiring high inspiratory flow rate. The rate required to deliver the medication in pMDIs is about 30 L/min while the rate required for DPIs is higher (ranging from 30 to 120 L/min) [29]. Furthermore, because of the wide range of DPI designs, healthcare educators often find challenges in developing and updating instructions regarding the inhaler technique. The current study revealed that the inhaler technique among asthmatic pregnant women is not an exception with regard to the prevalence and types of critical errors. Furthermore, as we have demonstrated in a previous study on asthma in general population in this country [26], there was a significant association between the inhaler technique and the patient's general knowledge about asthma medications as well as type of clinic in which asthma follow-up was conducted (respiratory vs. nonrespiratory clinics). Deficiency in asthma education and self-management skills is probably the most important reason to contribute to a poor inhaler technique during pregnancy. In addition, many pregnant subjects have concerns about the use of asthma medications during pregnancy and fear their effects on infant's well-being. This, in turn, may result in poor adherence and follow-up of their asthma during pregnancy time and hence poor asthma education including inhaler use [22]. The influences of various physiologic changes in respiratory function that happen during pregnancy on inhaler use are unknown, and it would be interesting to address such influences in future studies. The finding of high prevalence of inhaler errors in this study augments the international-guideline suggestions that management of asthma during pregnancy should be multidisciplinary. Several healthcare professionals should coordinate asthma management during pregnancy. These include nurses, asthma educators, pharmacists, midwives, and primary care physicians, as well as respiratory specialists and obstetricians [42, 43]. An interesting finding in this study is the lack of association between the inhaler technique and asthma control. This finding should be interpreted with caution as it may have been due to the fact that the majority of the study participants (65.3%) were categorized as poorly controlled asthma. Furthermore, we recently identified multiple other barriers to asthma control in this cohort of pregnant women [22]. Lack of a significant association between the inhaler technique and asthma control may simply reflect the significant contribution of such barriers to poor asthma control. Similar finding of lack of association between inhaler technique and asthma control due to the presence of multiple other reasons for poor asthma control was also reported from Jordan [44]. In a study from Malaysia, Loh et al. assessed the relationship between technique efficiency and frequency of daily short-acting *β*2-agonist (SABA) use and asthma exacerbations over a 12-month period [45]. There were no significant differences between the efficient and inefficient inhaler users in relation to frequency of daily SABA use or asthma exacerbations over the study period. The lack of difference was attributed to other factors such as patient's compliance to treatment, adequacy of treatment with maintenance inhaled corticosteroids (ICS), differences in asthma phenotype, and differences in perception of dyspnea among asthmatic patients [45]. Despite the lack of statistical significance concerning the association between the inhaler technique and maternal and fetal outcomes in the current study, one could observe a trend towards an association with regard to certain outcomes such as development of any maternal complication, rate of cesarean section, and development of asthma symptoms during delivery. A caution should be exercised when interpreting the link between pregnancy outcomes and the inhaler technique due to the small size of the study population. To the best of our knowledge, the current study is among the first to address errors in the inhaler technique in pregnant asthmatic women. It shed the light on an important aspect of asthma care in pregnant women related to medication use. In this study, patients' interview was conducted face-to-face by trained physicians with good background knowledge of asthma and asthma guidelines who were not responsible for asthma care of the participants. This has permitted direct observation of the inhaler technique by the interviewing physician.

### 4.1. Limitations of the Study

Besides the small number of participants, an important limitation of this study is the reliance of physician diagnosis of asthma due to the underuse of spirometry in this country. This could result in an impure asthma cohort. Furthermore, the cross-sectional design of the study permitted the inhaler technique and asthma control assessment only at a single point in time during pregnancy and hence may not reflect the whole figure throughout pregnancy. As asthma control is the major asthma-related determinant of fetal and maternal outcomes in asthmatic pregnant women, the finding of lack of associations between the inhaler technique and maternal and fetal outcomes in the current study may simply reflect the lack of association between the inhaler technique and asthma control. Finally, there are wide discrepancies within the literature regarding definitions and descriptions of inhaler errors and their classification as either “critical” or “noncritical.” This heterogeneity and lack of consensus could be a contributing factor to extremely different conclusions even with the same inhaler device type [46].

## 5. Conclusions

Improper inhalation technique is significantly prevalent in pregnant asthmatic women, particularly among those being followed in nonspecialized respiratory clinics and those with poor asthma education. The lack of significant association between the inhaler technique and asthma control (and hence maternal and fetal outcomes) may simply reflect the high prevalence of uncontrolled asthma and significant contribution of other barriers to poor asthma control in the current patient's cohort. Multidisciplinary management of asthma during pregnancy with particular emphasis on asthma and medication education is highly needed.

## Figures and Tables

**Table 1:** Patient's characteristics.

Age (years) (*n*=80)	
Mean	31.6 ± 6.2
Median	32
Age at onset of asthma (*n*=78)	
Mean	13.3 ± 11.1
Median	12.5
Educational level (*n*=77)	
Elementary and primary	5 (6.5%)
Secondary and above	72 (93.5%)
Total number of pregnancies (*n*=75)	
One pregnancy	12 (16%)
Two pregnancies	15 (20%)
Three or more pregnancies	48 (64%)
Asthma control using ACT (*n*=75)	
Uncontrolled	49 (65.3%)
Controlled	26 (34.7%)

**Table 2:** Errors in inhaler use.

Wrong inhaler step	No. of patients (%)^*∗*^
Errors in MDI use (no. = 47)	
Removing cap and shaking the inhaler	15 (31.9)
Breathing out gently before using the inhaler	29 (61.7)
Putting the mouthpiece in the mouth and applying good seal with the lips	3 (6.4)
Pressing the canister down at start of inspiration and continuing to inhale slowly and deeply (coordination between inhalation and generation of the aerosol)	32 (68.1)
Performing a breath-hold for at least 10 seconds	7 (14.9)
Errors in DPI use (no. = 20)	
Breathing out gently before using the inhaler	11 (55)
Placing the inhaler in the mouth and creating an adequate seal with the lips	5 (25)
Deep and forceful inhalation of the powder	5 (25)
Removing the inhaler from the mouth and performing a breath-hold for 10 seconds	13 (65)

^*∗*^Some patients performed more than one wrong step.

**Table 3:** Association between inhaler technique and different factors.

Variable	Correct inhaler use (*N*)	Incorrect inhaler use (*N*)	*P* value
Patient's knowledge about types of asthma medications			**0.002**
Does not know the difference between reliever and controller	9	34	
Knows the difference between reliever and controller	17	12	
Type of asthma follow-up clinic			**0.030**
Respiratory clinic	10	7	
Not respiratory clinic	16	40	
Duration of asthma			**0.501**
1–10 years	7	15	
11–20 years	10	10	
>20 years	9	22	
Age of asthma onset			**0.975**
15 years or less	14	25	
>15 years	10	20	
Nationality			**0.751**
Qatari	8	18	
Arab	11	14	
Asian	5	13	
Others	2	2	
Patient's level of education			**0.114**
High school or less	15	20	
University or higher	10	26	
Monthly income			**0.814**
Up to 10,000 QR	4	14	
10001–20,000 QR	11	14	
>20,000 QR	3	4	
Total number of pregnancies (gravida)			**0.257**
One pregnancy	1	10	
Two	5	10	
Three or more	17	26	
Asthma control using ACT			**0.619**
Controlled	9	15	
Uncontrolled	14	31	
Type of DPI used			**0.000**
Seretide Diskus	11	15	
Symbicort Turbuhaler	9	4	
Budesonide Turbuhaler	1	1	

**Table 4:** Association between inhaler technique and maternal and fetal outcomes.

Outcome	Correct inhaler use	Incorrect inhaler	*P* value
Any maternal complication of pregnancy			**0.542**
No any maternal complication	15	16	
Development of any maternal complication	9	21	
Cesarean section			**0.560**
Cesarean section	10	20	
Normal vaginal delivery	11	15	
Development of asthma symptoms during delivery			**0.109**
No asthma symptoms during delivery	15	18	
Asthma symptoms during delivery	8	20	
Any infant complication (respiratory distress/congenital anomalies)			**0.641**
No infant complication	17	30	
Development of any infant complication	7	8	
Low birth weight			**0.278**
Normal birth weight	17	23	
Low birth weight	1	7	

## Data Availability

The data used to support the findings of this study are available from the corresponding author upon request.

## References

[1] Global Initiative for Asthma (2017). Global strategy for asthma management and prevention. http://www.ginasthma.com.

[2] Kwon H. L., Belanger K., Bracken M. B. (2003). Asthma prevalence among pregnant and childbearing-aged women in the United States: estimates from national health surveys. *Annals of Epidemiology*.

[3] Hansen C., Joski P., Freiman H. (2012). Medication exposure in pregnancy risk evaluation program: the prevalence of asthma medication use during pregnancy. *Maternal and Child Health Journal*.

[4] Fitzsimons R., Greenberger P., Patterson R. (1986). Outcome of pregnancy in women requiring corticosteroids for severe asthma^*∗*^. *Journal of Allergy and Clinical Immunology*.

[5] Perlow J. H., Montgomery D., Morgan M. A., Towers C. V., Pronto M. (1992). Severity of asthma and perinatal outcome. *American Journal of Obstetrics and Gynecology*.

[6] Schatz M., Zeiger R. S., Hoffman C. P. (1990). Intrauterine growth is related to gestational pulmonary function in pregnant asthmatic women. *Chest*.

[7] Demissie K., Breckenridge M. B., Rhoads G. G. (1998). Infant and maternal outcomes in the pregnancies of asthmatic women. *American Journal of Respiratory and Critical Care Medicine*.

[8] Capstick T. G., Clifton I. J. (2014). Inhaler technique and training in people with chronic obstructive pulmonary disease and asthma. *Expert Review of Respiratory Medicine*.

[9] Newman S. P. (1985). Aerosol deposition considerations in inhalation therapy. *Chest*.

[10] Everard M. L. (2001). Guidelines for devices and choices. *Journal of Aerosol Medicine*.

[11] Aggarwal B., Gogtay J. (2014). Use of pressurized metered dose inhalers in patients with chronic obstructive pulmonary disease: review of evidence. *Expert Review of Respiratory Medicine*.

[12] Giraud V., Allaert F.-A., Roche N. (2011). Inhaler technique and asthma: feasability and acceptability of training by pharmacists. *Respiratory Medicine*.

[13] Melani A. S., Bonavia M., Cilenti V. (2011). Inhaler mishandling remains common in real life and is associated with reduced disease control. *Respiratory Medicine*.

[14] Lewis A., Torvinen S., Dekhuijzen P. N. (2016). The economic burden of asthma and chronic obstructive pulmonary disease and the impact of poor inhalation technique with commonly prescribed dry powder inhalers in three European countries. *BMC Health Services Research*.

[15] SIGN 153 (2016). *British Guideline on the Management of Asthma. A National Clinical Guideline*.

[16] Global Initiative for Asthma (GINA) (2007). Patient guide. http://www.ginasthma.org/local/uploads/content/files/GINA_PatientGuide2007/.

[17] Melzer A. C., Ghassemieh B. J., Gillespie S. E. (2017). Patient characteristics associated with poor inhaler technique among a cohort of patients with COPD. *Respiratory Medicine*.

[18] Allen S. C., Prior A. (1986). What determines whether an elderly patient can use the meter-dose inhaler correctly?. *British Journal of Diseases of the Chest*.

[19] Williams M. V., Baker D. W., Honig E. G., Lee T. M., Nowlan A. (1998). Inadequate literacy is a barrier to asthma knowledge and self-care. *Chest*.

[20] Basheti I., Bosnic-Anticevich S., Armour C., Reddel H. (2013). Checklists for dry powder inhaler technique: a review and recommendations. *Respiratory Care*.

[21] Price D. B., Roman-Rodriguez M., McQueen R. B. (2017). Inhaler errors in the CRITIKAL study: type, frequency, and association with asthma outcomes. *Journal of Allergy and Clinical Immunology: In Practice*.

[22] Ibrahim W. H., Rasul F., Ahmad M. (2018). Asthma knowledge, care, and outcome during pregnancy: the QAKCOP study. *Chronic Respiratory Disease*.

[23] Palmsten K., Schatz M., Chan P. H., Johnson D. L., Chambers C. D. (2016). Validation of the pregnancy asthma control test. *Journal of Allergy and Clinical Immunology In Practice*.

[24] Araujo G. V., Leite D. F., Rizzo J. A., Sarinho E. S. (2016). Asthma in pregnancy: association between the asthma control test and the global initiative for asthma classification and comparisons with spirometry. *European Journal of Obstetrics and Gynecology and Reproductive Biology*.

[25] American Thoracic Society (2017). *Asthma Control Test*.

[26] Ibrahim W. H., Suleiman N. N., El-Allus F. (2015). The burden of adult asthma in a high GDP per capita country: the QASMA study. *Annals of Allergy, Asthma and Immunology*.

[27] Leach C. L., Davidson P. J., Boudreau R. J. (1998). Improved airway targeting with the CFC-free HFA-beclomethasone metered-dose inhaler compared with CFC-beclomethasone. *European Respiratory Journal*.

[28] Sanchis J., Corrigan C., Levy M., Viejo J. (2013). Inhaler devices–from theory to practice. *Respiratory Medicine*.

[29] Laube B. L., Janssens H. M., de Jongh F. H. (2011). What the pulmonary specialist should know about the new inhalation therapies. *European Respiratory Journal*.

[30] Pauwels R., Newman S., Borgstrom L. (1997). Airway deposition and the airway effects of antiasthma drugs delivered from metered-dose inhalers. *European Respiratory Journal*.

[31] Newman S. P., Pavia D., Clarke S. W. (1981). How should a pressurized *β*-adrenergic bronchodilator be inhaled?. *European Journal of Respiratory Diseases*.

[32] Lawford P., McKenzie D. (1983). Pressurized aerosol inhaler technique: how important are inhalation from residual volume, inspiratory flow rate and the time interval between puffs?. *British Journal of Diseases of the Chest*.

[33] Newman S. P., Pavia D., Garland N., Clarke S. W. (1982). Effects of various inhalation modes on the deposition of radioactive pressurized aerosols. *European Journal of Respiratory Diseases. Supplement*.

[34] Everard M. L., Devadason S. G., Summers Q. A., Le Souef P. N. (1995). Factors affecting total and “respirable” dose delivered by a salbutamol metered dose inhaler. *Thorax*.

[35] Buttini F., Miozzi M., Balducci A. G. (2014). Differences in physical chemistry and dissolution rate of solid particle aerosols from solution pressurized inhalers. *International Journal of Pharmaceutics*.

[36] Levy M. L., Dekhuijzen P. N., Barnes P. J. (2016). Inhaler technique: facts and fantasies. A view from the aerosol drug management improvement team (ADMIT). *NPJ Primary Care Respiratory Medicine*.

[37] Thorsson L., Kenyon C., Newman S. P., Borgstrom L. (1998). Lung deposition of budesonide in asthmatics: a comparison of different formulations. *International Journal of Pharmaceutics*.

[38] Hardwell A. 1, Barber V., Hargadon T., McKnight E., Holmes J., Levy M. L. (2011). Technique training does not improve the ability of most patients to use pressurized metered-dose inhalers (pMDIs). *Primary Care Respiratory Journal*.

[39] Al Ammari M., Sultana K., Yunus F., Al Ghobain M., Al Halwan S. (2016). A cross-sectional observational study to assess inhaler technique in Saudi hospitalized patients with asthma and chronic obstructive pulmonary disease. *Saudi Medical Journal*.

[40] Sanchis J., Gich I., Pedersen S. (2016). Aerosol drug management improvement team (ADMIT). Systematic review of errors in inhaler use: has patient technique improved over time?. *Chest*.

[41] Crompton G. K. (1982). Problems patients have using pressurized aerosol inhalers. *European Journal of Respiratory diseases. Supplement*.

[42] Busse W. W. (2005). NAEPP expert panel reportmanaging asthma during pregnancy: recommendations for pharmacologic treatment—2004 update. *Journal of Allergy and Clinical Immunology*.

[43] Murphy V. E. (2015). Managing asthma in pregnancy. *Breathe*.

[44] Basheti I. A., Obeidat N. M., Ammari W. G., Reddel H. K. (2016). Associations between inhaler technique and asthma control among asthma patients using pressurized MDIs and DPIs. *International Journal of Tuberculosis and Lung Disease*.

[45] Loh L. C., Teng C. L., Teh P. N., Koh C. N., Vijayasingham P., Thayaparan T. (2004). Metered-dose inhaler technique in asthmatic patients - a revisit of the Malaysian scene. *Medical Journal of Malaysia*.

[46] Usmani O. S., Lavorini F., Marshall J. (2018). Critical inhaler errors in asthma and COPD: a systematic review of impact on health outcomes. *Respiratory Research*.

